# 

**Published:** 1841-10-01

**Authors:** 


### BIBLIOGRAPHICAL RECORD.

1. Memoranda on France, Italy, and Germany, with Remarks on Climates, Mecal Practice, Mineral Waters, &c. &c. By Edwin Lee, Esq. M.R.C.S. Octavo, pp. 342. Saunders and Otley, London, 1841.

---

2. A Practical Essay on some of the principal Surgical Diseases of India. By F. H. Brett, Esq. M.R.C.S. Bengal Medical Service, &c. Octavo, pp. 507. With numerous Plates. Calcutta, W. Thacker and Co. 1840.

---

3. The India Journal of the Medical and Surgical Sciences, Dec. 1840.

---

4. The India Review of Foreign Science and the Arts, Dec. 1840.

---

5. The Retrospect of Practical Medicine and Surgery, being a half-yearly Journal, &c. By W. Braithewaite, M.R.C.S. Surgeon to the Leeds General Eye and Ear Infirmary, &c. Vol. II. No. 3, January and June, 1841. Simpkin & Co. 1841.

☞ *This useful and laborious compilation or concentratian of practical medicine increases in value as it proceeds.*

---

6. A History of British Forest Trees. By Prideaux John Selby, F.R.S. &c. Illustrated by Woodcuts of each Species, and numerous Vignettes. Part I. Price 2s. 6d. July, 1841. J. Van Voorst, London.

---

7. A new Operation for the Cure of Amaurosis, Impaired Vision, &c. By James J. Adams, F.L.S. Price 2s. 6d. Churchill, July, 1841.

---

8. Note sur le Developpement de Nerfs Particuliers a la Surface du Cervervelet. Par le Docteur Bennett.

---

9. A Catalogue of Plants collected in the Neighbourhood of Banbury. By George Gulliver, F.R.S. Assistant-Surgeon to the Royal Regiment of Horse-Guards. Tilt and Bogue, London. July, 1841.

10. Provincial Medical and Surgical Journal. Edited by Drs. Green and Streeten. Published weekly.

---

11. The Dental Art. A practical Treatise on Dental Surgery. By Chapin A. Harris, M.D. Baltimore, 1839.

---

12. L'Examinateur Medical, Nos. 1 & 2. 1841.

---

13. Transactions of the Medical and Physical Society of Bombay, 1840.

---

14. A Letter to Sir B. Brodie, Bart., on the Application of the Collegiate System to the Medical Schools of the Metropolis. By the Rev. J. H. North, Chaplain to St. George's Hospital. Churchill & Hatchard. 1841.

---

15. An Address delivered to the Council of the College of Surgeons in London, on the occasion of quitting the Chair, July 8, 1841. By John Painter Vincent Wix. London, 1841.

---

16. Criminal Jurisprudence, in relation to Mental Organization. By M. B. Sampson. Highley, 1841.

---

17. Illustrations of Phrenology, comprising Accounts of Persons remarkable in some mental respect, &c. By G. R. Lewis. No. 1. Curvoisier. S. Highley, July, 1841.

---

18. Statistics of the Retreat, near York, from its Establishment in 1796 till 1840. By John Thurnam, Esq. Surgeon. York, July, 1841.

---

19. Medical Report of the Managers of the Lunatic Asylum of Aberdeen for the Year ending 30th April, 1841.

---

20. Copy—Report of Sir Alexander Morison, presented to the Quarterly Court of Governors, Jan. 1841.

---

21. Hippopathology, (Part II. completing Vol. II.) the Diseases of the Digestive, Urinary, and Generative Organs of the

Horse, with the different Methods of Castration, &c. By William Percivall, M.R.C.S. Pp. 257. With Plates. Longman and Co. 1841.

---

22. The Anatomy of the Arteries. By Richard Quain, M.D. Part VIII. Five Plates, with Letter-press, price 12s. The Delineations by Joseph Maclise, Esq. Surg. Taylor and Walton. July, 1841.

---

23. Annales de la Chirurgie Francaise et Etrangere. Par MM. Begin, Marchal, Velpeau, and Vidal. Nos. 7. and 8. Juillet and August, 1841. Bailliere, Regent Street, London.

---

24. Report of the Directors of the Montrose Lunatic Asylum Infirmary, and Dispensary, for the Year ending June 1, 1840.

---

25. Edinburgh Monthly Journal of Medical Science. Edited by J. Rose Cormac, M.D. No. VIII. Aug. 1841.

---

26. The Cyclopædia of Practlcal Surgery, &c. By W. Costello, M.D. Part IX. August, 1841.

---

27. The Naturalist's Library, &c. Mammalia. Vol. XI. Marsupialia or Pouched Animals. By G. R. Waterhouse, Esq. Edinb. Lizars, 1841.

---

28. Philosophic Nuts; or the Philosophy of Things, as developed from the Study of the Philosophy of Words. By Edward Johnson, Esq. Nos. 8 and 9. Simpkin and Co. Price one shilling each. August and September, 1841.

---

29. Normal Anatomy of the Liver. By Erasmus Wilson, Esq. (From the Cyclopædia of Anatomy and Physiology.) 1841.

☞ *Very ably executed.*

---

30. A Dictionary of Practical Medicine, &c. Part VII. By James Copland, M.D. F.R.S. Price 4s. 6d. Longman and Co. Sept. 1. 1841.

31. The Dublin Journal of Medical Science. No. 58. Sept. 1841.

---

32. The London and Edinburgh Journal of Medical Science. Edited by J. B. Cormack, M.D. London, Bailliere; Edinb. Machlachlan and Co. Price 1s. 6. No. IX. Sept. 1841.

---

33. Actes passés a la Seconde Session de la Quatorzieme Legislature de l'Etat de la Louisiane, &c. Nouvelle Orleans, 1840.

---

34. Memoir of the Case of a Gentleman born Blind, and successfully operated on in the 18th year of his age; with Physiological Observations and Experiments. By J. C. August Franz, M.D. (From the Philosophical Transactions.)

---

35. A Practical Treatise on the Efficacy of Mineral Waters in the Cure of Chronic Diseases. Illustrated by Cases. With an Appendix, containing Analyses of the most reputed Spas in Germany. By Sir Alex. Mackenzie Downie, M.D. &c. &c. Small 8vo, pp. 219. Frankfort—and Churchill, London.

☞ *A concise and sensible little Treatise.*

---

36. A short Description of Kissengen—its Baths and Mineral Waters. By Dr. F. A. Balling. Translated into English by Sir A. M. Downie, M.D. Small octavo, pp. 44. Frankfort, Jugel. London, Churchill, 1841.

---

37. The Oration, delivered before the Medical Society of London, at the 68th Anniversary, March 8th, 1841. By W. D. Chowne, M.D. &c.

---

38. A Practical Treatise on the New Operation for lateral Curvatures of the Spine, &c. With Plates. By G.B. Childs, M.R.C.S. Quarto, pp. 56. Whittaker, London, 1841.

